# Development and Evaluation of a Disease Large Animal Model for Preclinical Assessment of Renal Denervation Therapies

**DOI:** 10.3390/ani10091446

**Published:** 2020-08-19

**Authors:** Armando Pérez de Prado, Claudia Pérez-Martínez, Marta Regueiro-Purriños, Carlos Cuellas-Ramón, María López-Benito, José Manuel Gonzalo-Orden, Rodrigo Estévez-Loureiro, Ana Isabel Cortina-Rivero, David Viñuela-Baragaño, José R Altonaga, Armando Tellez, Felipe Fernández-Vázquez

**Affiliations:** 1 Fundación Investigación Sanitaria de León, Hospital Universitario de León—Instituto de Biomedicina (IBIOMED), Universidad de León, 24071 León, Spain; aperez@secardiologia.es (A.P.d.P.); mregf@unileon.es (M.R.-P.); ccuellas@secardiologia.es (C.C.-R.); merislobe@hotmail.com (M.L.-B.); manolo@unileon.es (J.M.G.-O.); roiestevez@hotmail.com (R.E.-L.); anabelcortinarivero@gmail.com (A.I.C.-R.); dvinb@unileon.es (D.V.-B.); jarodma@unileon.es (J.R.A.); ffernandezv@saludcastillayleon.es (F.F.-V.); 2 Alizée Pathology LLC., Thurmont, MD 21788, USA; armando.tellezcruz@outlook.com; 3 Cátedra de Cardiología y Medicina Vascular, Escuela de Medicina, Tecnológico de Monterrey, 64710 Monterrey, Mexico

**Keywords:** hypertension, DOCA, renal denervation, radiofrequency, swine, histology, immunohistochemistry

## Abstract

**Simple Summary:**

High blood pressure (arterial hypertension) is a major public health problem as far as it has a high prevalence in the adult population and it entails significant cardiovascular and cerebrovascular risks. New ways of treating hypertension include catheter-based renal denervation, i.e., modulation of the autonomic nervous system that has a crucial role in the regulation of blood pressure. We have designed a hypertensive animal model to test the efficacy and safety of this kind of treatment. After the induction of mineralocorticoid mediated hypertension in minipigs, we have analyzed the durability of hypertensive state, the response to drugs and the effects of renal denervation obtained with a radiofrequency catheter (Symplicity^®^, Medtronic, Santa Rosa, CA, USA). The attrition rate was high, mainly caused by infections in this instrumentalized animal model. The effect of renal denervation was confirmed by pathology (nerve damage), biochemical parameters and blood pressure measurements. The conclusion is that this model could be used in the assessment of different therapies aimed to modulate the influence of the autonomic nervous system on hypertension if the high mortality rate is reduced with a less invasive monitorization system.

**Abstract:**

New-generation catheters-based renal denervation (RDN) is under investigation for the treatment of uncontrolled hypertension (HTN). We assessed the feasibility of a large animal model of HTN to accommodate the human RDN devices. Ten minipigs were instrumented to measure blood pressure (BP) in an awake-state. HTN was induced with subcutaneous 11-deoxycorticosterone (DOCA, 100 mg/kg) implants. Five months after, the surviving animals underwent RDN with the Symplicity^®^ system. Norepinephrine (NE) renal gradients were determined before and 1 month after RDN. Renal arteries were processed for histological (hematoxylin-eosin, Movat pentachrome) and immunohistochemical (S100, tyrosine-hydroxylase) analyses. BP significantly rose after DOCA implants. Six animals died prematurely, mainly from infectious causes. The surviving animals showed stable BP levels after 5 months. One month after RDN, nerve damage was showed in three animals, with impedance drop >10%, NE gradient drop and reduction in BP. The fourth animal showed no nerve damage, impedance drop <10%, NE gradient increase and no change in BP. In conclusion, the minipig model of DOCA-induced HTN is feasible, showing durable effects. High mortality should be addressed in next iterations of this model. RDN may partially offset the DOCA-induced HTN. Impedance drop and NE renal gradient could be markers of RDN success.

## 1. Introduction

Uncontrolled hypertension (HTN) has been described as a multifactorial phenomenon involving multiple biological mechanisms; the hyperactivity of the sympathetic nervous system plays a paramount role in its onset, maintenance and progression [1]. To date, multiple procedures for renal nerve ablation have been studied, amongst them transcatheter renal radiofrequency (RF) ablation of the afferent and the efferent nerves remains the most commonly investigated technique in preclinical [2,3] and human studies [4,5]. However, the field of renal denervation (RDN) experienced a setback when the results of the SYMPLICITY HTN-3 trial were presented [6]. The reasons for the failure of SYMPLICITY HTN-3 are multiple, including incomplete denervation, inexperience of the operators, improper selection of patients, and imperfect design of the study itself [7]. Therefore, new preclinical investigations could help to improve and redesign the delivery techniques.

In this preclinical setting, large-scale studies have been performed to determine the safety of delivering RF therapy to the renal nerves. Immediate [8] and chronic [9] tissue effects following RF ablation have been described. Even the nerve responses following the ablation therapies have been carefully explored [10]. However, is important to note that while these therapies specifically targeted for patients labeled as “resistant hypertensive” patients, the preclinical evaluation of every renal denervation device in the premarket stage, as well as the ones commercially available, has been tested in healthy, normotensive animals. While we agree that the norepinephrine quantification and the histopathological assessment provides an accurate panorama of the effects of this therapy, we believe that there is, without a doubt, room in the experimental setting for a more accurate platform that would mimic the clinical challenges. These studies are considered paramount to guide future clinical studies and interventions [11]. Unfortunately, an ideal animal model for RDN study is still unavailable. To date, some large animal models of HTN have been proposed to clarify pathophysiological mechanisms of RDN, with variable success [12,13,14,15].

In this study, our goal is to develop a large animal model of hypertension that would be reliable, reproducible, and affordable to industry and academia and that would accurately represent the clinical scenario. We analyze the feasibility of a minipig model of 11-deoxycorticosterone (DOCA)-induced HTN, assessing the persistence of HTN and the effect of RDN on the DOCA-induced HTN. However, the high mortality should be addressed in next iterations of this model.

## 2. Materials and Methods

**Figure 1:**
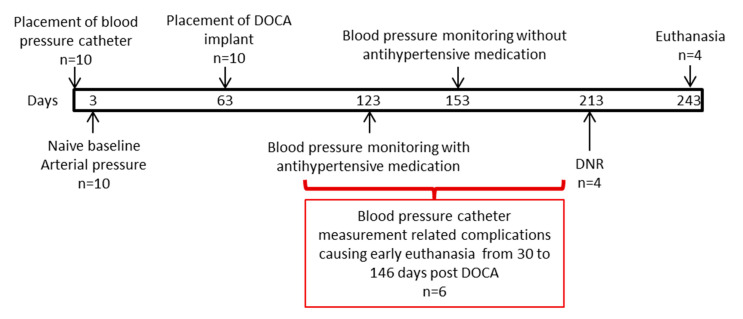
Scheme of study design.

The care and use of animals for this preclinical study was reviewed and approved by the Ethics Committee of the Consejería de Agricultura y Ganadería (01/06/15; Junta de Castilla y León, Spain) prior to the start of the study. The treatment of the animals was in accordance with relevant site procedures, which utilized the local regulations outlined in the RD 53/2013, 1 February, which defined basic standards for the protection of animals during experimentation and other scientific purposes, such as education, and European Directive 2010/63/EC and the conditions specified in The Guide for Care and Use of Laboratory Animals, Eighth Edition (ILAR publication, 2010, National Academy Press) as a guide. The study design is presented in Figure 1.

### 2.1. Animal Model

The study was implemented using ten 3-month-old female minipigs weighing 20 ± 3 kg, which were provided by Granja Experimental IMIDRA N Registro EX 013-C. The pigs, housed individually in a special room with a suitable temperature (23 ± 1 °C) and humidity (50 ± 5%), were maintained on a standard chow diet.

**Figure 2:**
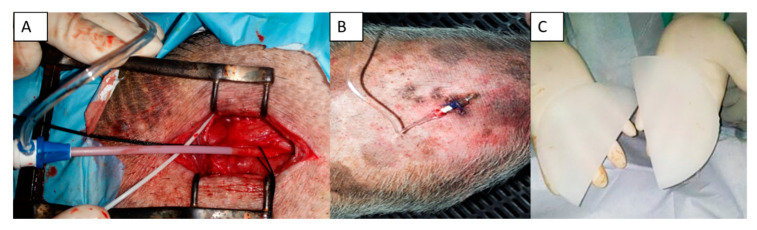
(**A**,**B**) The arterial blood pressure catheter was placed in all swine and subcutaneously tunneled out for easy access and monitoring; (**C**) Silicon-based 11-deoxycorticosterone implants that were placed in all swine for the creation of the hypertension model.

Following the acclimation period (at least 1 week), all animals underwent the placement of a blood pressure (BP) measurement catheter. On the day of catheter placement, the animals were administered 0.35 mg/kg of midazolam and 5 mg/kg of ketamine intramuscular. After the level of sedation was verified to be adequate, a dose (1–2 mg/kg) of anesthetic propofol was administered by slow intravenous infusion. It was inserted through an endotracheal tube and connected the animals to volume-controlled mechanical ventilation, maintaining anesthesia with sevoflurane at 1.5–2%. Analgesia was procured with intramuscular buprenorphine 0.01 mg/kg. The permanent intra-arterial catheter (Drucafix, B. Braun, Melsungen, Germany) was positioned initially in the carotid artery; however, catheters were placed in other locations and sometimes moved to the contralateral location or remotely (femoral artery) to ensure proper arterial access. The catheter was tunneled subcutaneously, proximally to an external location that could be easily accessible to the staff and not accessible to the animals to produce damage to the catheter. Following the animal after recovery, multiple BP reads per day over a period of 3 days were acquired while the animal was awake. This measurement was considered baseline for the animal. For a period of 60 days, the BP was monitored and averaged (Figure 1 and Figure 2A,B). After this initial period, all animals underwent surgical placement of a subcutaneous silicone-based implant that contained DOCA (100 mg/kg) (Figure 1 and Figure 2C). During the stage of the study, all animals received a mean of 10,560 mg of potassium supplements orally. The impact of the DOCA implants in BP was followed for 60 days without treatment. Following the effects of DOCA in BP, in order to evaluate the response of the hypertension obtained by the model creation, Spironolactone (up to 200 mg/day) and Doxazosin (up to 16 mg/day) were utilized. After a month of antihypertensive medication regimen, the Spironolactone and Doxazosin was stopped to determine if the hypertensive model effect was still present. Approximately 60 days after stopping the medication and with continued veterinary and BP monitoring, the animals received catheter-based ablation of the renal arteries. The RDN procedure is explained in detail below. All animals that received the RDN procedure were followed for 30 days after the RDN procedure. All animals received oral amoxicillin (plus clavulanic acid) 25 mg/kg for 15 days after every surgical procedure. If the animal presented gastrointestinal symptoms or prolonged duration of amoxicillin treatment, oral marbofloxacin (2 mg/kg daily) was administered.

### 2.2. Catheter-Based Renal Denervation Therapy Delivery

After 5 months of the initial hypertension induction procedure, RDN ablation therapy was performed according to common protocols of catheter-based renal denervation [12]. In brief, animals were fasted at least 12 h before the interventional procedure. Femoral arterial access was obtained under general anesthesia. All animals received 325 mg of Aspirin single dose daily for one day before the initial procedure. This same dose was administered on the day of the procedure and daily until the completion of the study. Anticoagulation with heparin was achieved during the procedure (100 Units/Kg IV) to maintain an activated clotting time ≥250 s. After evaluating the baseline angiography of the renal arteries, there were no renal arteries excluded based on anatomy (diameter less than 3.0 mm or greater than 7.0 mm, length less than 20 mm or amount of taper across target length greater than approximately 30%).

The Symplicity™ Renal Denervation System (Medtronic, Santa Rosa, CA, USA) is a highly flexible and 6 French guide catheter-compatible device with unique single point of contact energy delivery capabilities. Symplicity™ is designed to deliver low level RF energy through the wall of the renal artery for renal denervation. The system consists of two major components: a sterile single use ablation catheter and a proprietary generator, which delivers RF energy sufficient for ablating sympathetic nerves adjacent to the treatment site [16,17,18]. The RDN catheter was introduced and positioned in the right or left renal artery via fluoroscopic guidance. Bilateral RDN was initiated within each artery starting in a distal position. A standard treatment at the target location was performed using the following ablation settings: Power −8 W, Duration −120 s. Once the ablation was completed, the catheter was repositioned to locate the next ablation site approximately 5 mm proximal to the previous ablation site. Ablation was repeated enough times to completely cover and evenly distribute treatment sites among the length of the renal artery within the main stem and beyond distal bifurcation (“Y” therapy delivery) of the renal artery (branch arteries measuring at least 3.0 mm in diameter and at least 10 mm in length), avoiding the overlap of treatments. Nitroglycerin 200 mcg was injected directly into renal arteries prior to ablation to prevent vasospasm. After the procedure, the RF catheter was removed, and angiography was performed again to check the anatomical structure of the renal arteries. After final angiography, sevoflurane was discontinued and the animal was extubated when the gag reflex returned.

### 2.3. Gross Pathology and Tissue Harvesting

Complete necropsy and histopathological study were conducted on all animals that died during study to determine the cause of the death. At the end of the follow-up period, the animals were heparinized and humanely euthanatized. Gross pathology was performed in order to evaluate macroscopic changes to the treated kidneys, heart, lungs, liver, spleen, bowel, ovaries, adrenal glands prior to en-block resection of the treated renal arteries, attached kidneys and surrounding tissues.

For the extraction of the area of interest, the renal arteries were pressure perfused through the cannulated aorta with a Lactated Ringers solution followed by perfusion fixation with 10% neutral buffered formalin (NBF) solution for approximately 10 min. The treatment area and surrounding tissues that included the kidneys, major vessels, ureters, adrenal glands, renal lymph nodes and any associated soft tissues, were dissected. Tissue explants were immersed into 10% NBF for at least 2 days, supported by a solid cork backing to ensure stability and structural integrity of the tissue block. After fixation, the aorta was bisected to expose the renal ostia. The renal artery and approximately 1.5–2 cm radius of the surrounding retroperitoneal connective tissues was isolated by removing excess tissue to create a roughly 3-cm-wide renal “stump” centered on the renal artery. Equidistant sections were taken from the renal ostium to the renal hilum, resulting in 5–6 cross-sections along the length of the treated renal artery that included associated soft tissues (lymph nodes, soft connective tissue, veins, and nerves). These transverse sections were examined grossly for any evidence of injury.

### 2.4. Histological Examination

Tissues were processed routinely for histology and embedded in paraffin blocks. Serial 3 µm sections were stained with hematoxylin-eosin (HE) and Movat pentachrome stains. The histopathology evaluation of renovascular damage included: endothelial cell loss, medial damage (hyalinization and fibrosis), inflammation and adventitial fibrosis. The magnitude of the changes was assessed using a semiquantitative ordinal grading system: 0, none; 1, minimal; 2, mild; 3, moderate; 4, severe. The endothelial cell loss was evaluated by the percentage of circumferential endothelial cell loss and a semiquantitative score was established: 0, 100% of the luminal surface covered by endothelium; 1, endothelial loss <25%; 2, endothelial loss 25–50%; 3, endothelial loss 50–75% and 4, endothelial loss >75% of vessel circumference [19].

### 2.5. Immunohistochemistry

Sections were immunostained for expression of S100 for Schwann cells (1:1000 dilution, rabbit polyclonal Z0311, DakoCytomation, Glostrup, Denmark) and tyrosine hydroxylase (TH) for efferent nerve fibers (1:1000 dilution, ab41528, rabbit polyclonal Abcam, Cambridge, MA, USA). Serial 3 µm sections of paraffin-embedded samples were cut and mounted on poly-l-lysine-coated slides (Thermo Scientific, Germany). Sections were dewaxed in xylene and rehydrated in graded alcohol solutions, and they were stained using the Avidin–Biotin complex method or the EnVision(+) method for S100 or TH, respectively. Endogenous peroxidase activity was quenched by incubation in hydrogen peroxide (0.5%) solution in water for 30 min at room temperature. Tripsin 0.1% pH 7.8 (T7409 Sigma Aldrich, St Louis, MO, USA) or heat antigen retrieval (EDTA/TRIS Tween pH 9) was performed for S100 or TH, respectively. Following this procedure, slides were blocked with 5% goat serum (20 min, at 25 °C) and then incubated with primary antibodies (overnight, at 4 °C). The following day, slides were washed 3 times in TBS and incubated with a biotinylated secondary antibody [S100, biotinylated anti-rabbit (Santa Cruz Biotechnology, Inc., Dallas, TX, USA, 1:200 30 min) and TH, Envision polyclonal (K4003, Dako Glostrup, Denmark)]. Formalin-fixed, paraffin-embedded untreated renal artery sections were used as positive controls. The primary antibody was replaced with antibody diluent for negative controls. Labeling was visualized with application of 3-3-diaminobenzidine-tetrahydrochloride (DAB) as chromogen substrate (Peroxidase substrate Kit DAB SK-4100, Vector Laboratories, Burlingame, CA, USA). Slides were counterstained with Harris’s hematoxylin, dehydrated in graded alcohol, and mounted with coverslips. The intensity and distribution of immunostaining can be assessed using a semiquantitative scoring system: 0, no reaction; 1, patchy/very weak reaction; 2, weak to moderate reaction; 3, strong reaction. Grade 2 or lower was considered to be an indicator of nervous damage [19].

### 2.6. Morphometric Analysis

Morphometric analysis was conducted on the images using Image-Pro Plus software to quantify the following parameters: number of nerves per section (S100), number of damaged nerves per section (TH) and media thickness at and away from the RF contact point.

### 2.7. Norepinephrine Renal Gradient Determination

Blood samples were collected through the femoral catheter before RDN and 4 weeks after RDN. Arterial and venous blood samples were kept frozen until analysis at the bioanalytical test site (Laboratorio Echevarne, Barcelona, Spain). The entire volume of the blood samples was homogenized with 3/16″ stainless steel ball bearings in a bead homogenizer. Samples were centrifuged and filtered. Calibration standards were prepared in aqueous 10 mg/mL sodium metabisulfite and the calibration range was 0.100 to 100 ng/mL. Stable isotope labeled norepinephrine was added to each sample (standards, QCs and study samples) as an internal standard. Samples were derivatized followed by solid phase extraction. Samples were then injected onto an HPLC-MS/MS system for quantitation. Analysis was performed under HPLC with an electrochemical (potential amperometry).

### 2.8. Statistical Analyses

Values are expressed as percentages and as mean ± standard deviation, depending on the type of variable. Differences between means and repeated measurement of values were analyzed using the Student t test and analysis of variance. For multiple comparisons, a post hoc analysis was conducted using the Dunnett method for comparison with the control. Semiquantitative variables were analyzed using the chi-square test or the Fisher exact method. All analyses were conducted with the JMP v13 statistical software package (SAS Institute Inc., Cary, NC, USA) using a *p* value < 0.05 as a cutoff for statistical significance.

## 3. Results

### 3.1. Blood Pressure in DOCA Model and Effect of RDN Procedure

Ten female minipigs were included in this study. In all ten, the BP monitoring catheter was placed and an average baseline arterial pressure was possible to obtain. The DOCA implant was successful in all 10 animals. Following this procedure, the catheter needed to be relocated in multiple occasions due to occlusions or signs of infection at the access sites. Unfortunately, due to catheter complications, six animals resulted on early death or required early euthanasia following veterinary evaluation. Four animals survived to the end of the study. The causes of death were varied: for one animal, during the placement of the BP monitoring, the thoracic aorta was damaged, resulting in an aortic aneurysm and eventual rupture since the animal had a hypovolemic shock that was not resolved. Two animals developed septicemia that was not resolved despite antibiotic regimen, while the other developed endocarditis and valvular vegetations with associated thromboembolism. Two animals were euthanized due to impaired clinical status and one diagnosed with hypovolemia following upper gastrointestinal bleeding (gastric ulcer), and, for another, the source of the loss of blood was not found.

The early deaths and early euthanasia animals survived 87.5 ± 49.3 days post DOCA implant placement (from 30 to 146 days). As these animals survived model creation (*n* = 6) and some even survived to the initial antihypertensive medication regimen (*n* = 4), the BPs acquired during hemodynamically stable periods were still used in each according stage. The renal arteries from these animals (*n* = 12) were still harvested per protocol and evaluated as “non-RDN treated controls” for a total of 20 renal arteries evaluated.

**Figure 3:**
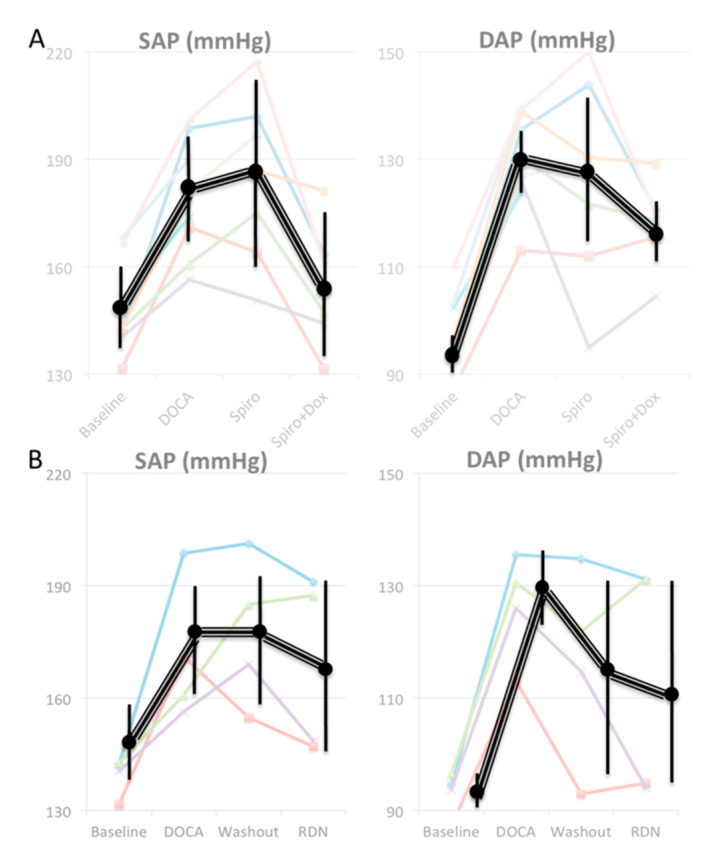
Systolic (SAP) and diastolic (DAP) arterial pressures in a minipig model of 11-deoxycorticosterone (DOCA)-induced hypertension (HTN). (**A**) Effect of the antihypertensive medication (*n* = 6). (**B**) Effect of the denervation renal (DNR) (*n* = 4).

The baseline systolic arterial pressure (SAP) was possible to acquire in all ten animals. The baseline SAP was 148 ± 12 mmHg (*n* = 10 animals). One month after the surgical placement of the DOCA implant, there was a 20% increase in SAP (179 ± 16 mmHg, *n* = 10 animals; *p* = 0.027). The values of diastolic arterial pressure (DAP) followed a similar pattern with baseline values of 98 ± 7 that raised to 130 ± 9 mmHg (*p* = 0.0001). Following the antihypertensive medication regimen (spironolactone 200 mg daily + doxazosine up to 16 mg daily), there was a significant decrease both in SAP (155 ± 7 mmHg, *n* = 6 animals; *p* = 0.019) and in DAP (115 ± 7 mmHg, *n* = 6 animals; *p* = 0.017) (Figure 3A). Following a “washout” period without antihypertensive medication and prior to the RDN treatment, it was evident that the animal model remained in its hypertensive state since there was subsequent increase in SAP similar to the pressure prior to the administration of antihypertensive medication (179.0 ± 18.4 mmHg, *n* = 4). One month after the RDN procedure, there was an evident decrease of approximately 6% (169.0 ± 22.7 mmHg, *n* = 4). Three animals showed a reduction ≥ 8 mmHg in SAP, whereas one pig (# 3) showed a slight increase in SAP. The response of DAP to RDN tended to be much more irregular. Figure 3B shows systolic and diastolic arterial pressures of denervated animals in different points of the study.

### 3.2. Angiography

The main renal arteries had an average length of 26.9 ± 9.7 mm between the aorta and the main arterial bifurcation. At baseline, the mean luminal diameter (LD) in the main renal artery was 5.3 ± 0.6 mm (left 5.5 ± 0.7 mm; right 5.1 ± 0.6 mm). Past the bifurcation, the LD of the branches was 3.5 ± 0.4 mm; the inferior renal branch being on average slightly larger (3.7 ± 0.3 mm) than the superior branch (3.3 ± 0.5 mm). The characteristic image previously described as “notches” following RF treatment delivery was evident in all treated arteries. The notches observed following treatment delivery at baseline were completely resolved at follow up. All arteries displayed undisturbed blood flow pre and post treatment delivery with no evidence of thrombus or intimal dissection.

### 3.3. Gross and Histological Evaluation

There was no evidence of collateral RF damage to any abdominal viscera following the catheter-based procedure. At gross evaluation, there were no abnormal changes noted in any of the renal arteries. There were no abnormal gross or microscopic pathological changes observed in the renal parenchyma of any kidneys that could be related to the delivery of RF therapy.

**Figure 4:**
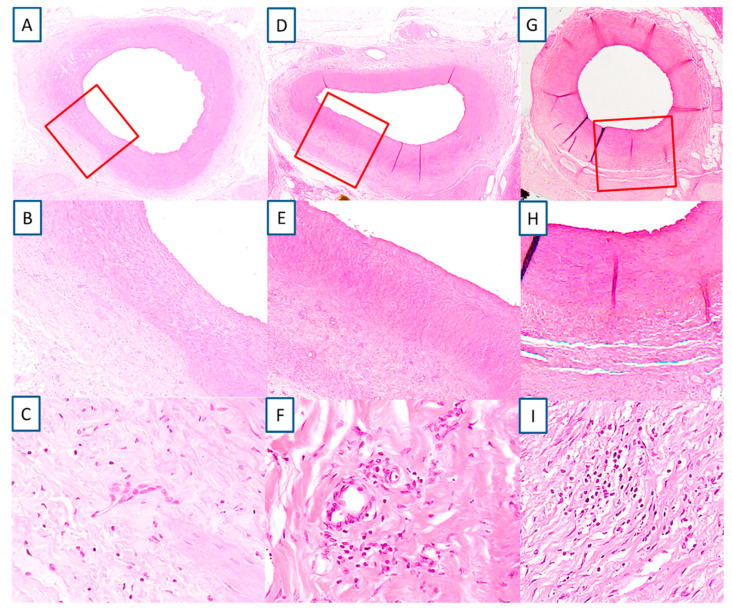
Characteristic features of a renal artery and arterial wall following radiofrequency ablation. The extent of fibrous tissue formation in the media and adventitia, the hyalinization of the media and low degree of inflammatory infiltration and complete recovery of the endothelium with lack of luminal thrombus. Animal # 1 (**A**–**C**); # 2 (**D**–**F**); # 4 (**G**–**I**). Hematoxylin-eosin.

**Figure 5:**
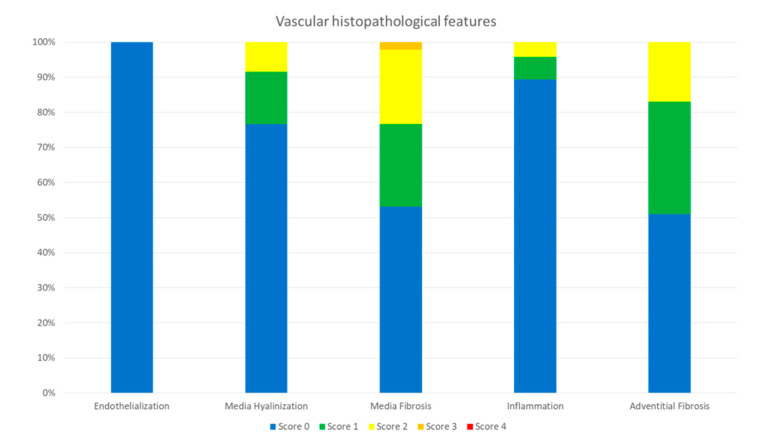
Vascular histopathological features in a DOCA minipig model after radiofrequency catheter-based renal denervation.

A total of 47 sections were examined from four animals with eight renal arteries. The location where RF treatment was delivered was evident in the exposed artery by the involvement of the media (Figure 4A,B,D,E,G,H). The media changes were confined to a well delineated area. RF tissue changes extended beyond the focal, medial treatment delivery areas to within perivascular tissues. The medial layer showed normal thickness at the point of RF therapy delivery (455.1 ± 204.2 µm) similar to the opposite unexposed wall (462.4 ± 103.4 µm). There was very minimal inflammation recorded, only 10.6% from the evaluated slides of the treated renal arteries showed presence of chronic inflammation, consisting primarily of lymphocytes, and even then the inflammation was low grade (score 1 and 2) (Figure 4C,F,I). There was minimal evidence of necrosis with histological features of healing such as media fibrosis and hyalinization and adventitial fibrosis (score 1 and 2). All vessels showed complete endothelialization (score 0, Figure 5). There were no mural thrombus, hemorrhage or hemosiderin deposit in the vessel wall.

**Figure 6:**
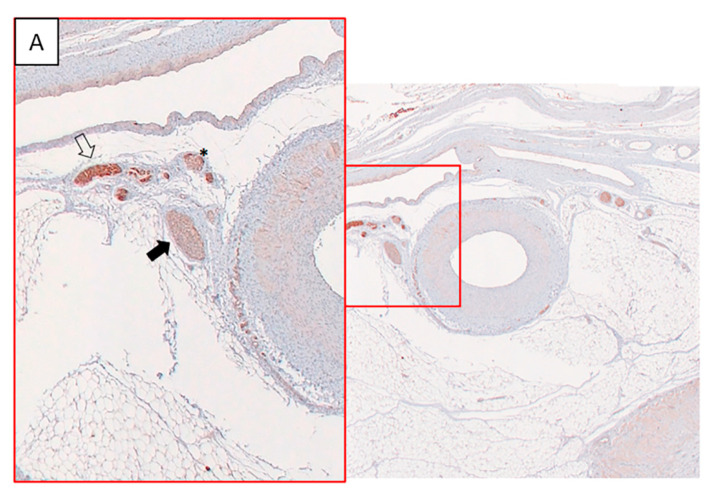

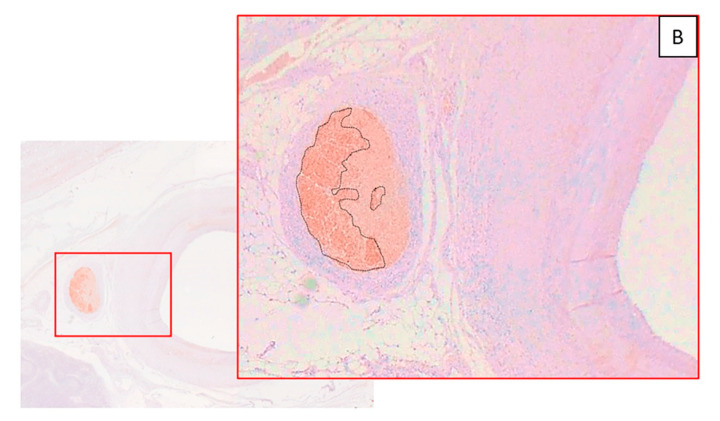
The images show representative images of the immunohistochemical staining with tyrosine hydroxylase (TH). (**A**) Three different types of affection following radiofrequency ablation. The clear arrow shows a nerve that has not yet been ablated at that level. The solid arrow shows evidence of a proximally ablated nerve displaying low TH staining demonstrating nerve atrophy. However, the nerve labeled with an asterisk shows how because of the nerve network, heterogeneous staining is displayed around some nerves with patches of strong TH staining combined with atrophied fibers. (**B**) A more evident example of this fiber heterogeneity (viable nerve in dotted line).

The percentage of damaged nerves per level was high in the animals # 1, 2 and 4 based on TH immunostaining (score ≤ 2), while almost all nerves showed normal appearance with TH staining = 3 in the animal # 3 (Figure 6A,B, Table 1).

### 3.4. Norepinephrine Gradient Results and Procedural Characteristics

A decrease in NE renal vein–artery difference (gradient) from pre to post procedural levels was observed in three animals (# 1, 2 and 4). Conversely, the animal # 3 showed an increase in this value. Interestingly, this animal didn’t show nerve damage at the pathological analysis. The NE gradient reduction showed a correlation with the impedance drop and the mean temperature achieved (Table 2).

## 4. Discussion

In this study, the hypertensive pig model induced by low dose sustained-release DOCA implantation is raised as a feasible platform to evaluate renal denervation therapies. The main findings of this study were as follows: (1) the minipig model of DOCA-induced HTN seems to be feasible, showing durable effects; (2) the RDN with RF may partially offset the DOCA-induced HTN after 4 weeks due to substantial nerve damage; (3) a relation may be established between the effectiveness of RF RDN (nerve damage, BP reduction) and procedural parameters (impedance drop, NE gradient); (4) the catheter-based RF RDN seems to be safe and only mild renal injury is observed; (5) the direct arterial BP monitoring through an indwelling catheter was unfeasible.

Disease animal models have been extensively used in the understanding of arterial hypertension [20]. The development of hypertension using DOCA in animal models is a concept that began in the late 1960s [21,22] and kept evolving in the 1980s [23] up to now [24]. Great experience of our scientific colleagues has been provided based on small preclinical models of hypertension (rodent models) [23,25,26,27]. Since large animal models provide the capability of similar anatomy and biodistribution compared to humans, some species have been utilized for the development of hypertension [12,14,15,28]. The swine is the commonly accepted animal model for catheter-based renal denervation device evaluation to due to providing the advantage of similar peripheral vasculature, the use of real size clinical equipment, vast experience in the histopathological evaluation as well as similar vascular and neurological responses to human [29]. In the present study, the surgical placement of the DOCA implant was carried out without complications. We have previously conducted a pilot study of mid-term (1–3 months) feasibility of DOCA implantation and indwelling catheter to monitor BP in Large White swine and minipigs without detecting significant infectious or thrombotic complications (data not shown). Following the subcutaneous implantation of DOCA, an evident increase (20%) in SAP was detected (*n* = 10 pigs), which is in line with previous reports [30,31]. More importantly, this model correctly responded to pharmacologic antihypertensive regimen with an evident decrease in systolic pressure (11%). Furthermore, the “washout” period without antihypertensive medication reiterated that this blood pressure decrease was related to the medication and the hypertensive background was still present for at least 5 months, displaying a subsequent increase in SAP similar to the pressure prior to the administration of antihypertensive medication. It is important to emphasize the antihypertensive drug utilized in our study. Doxazosin, a quinazoline compound, is a α1 adrenergic receptor blocker that inhibits the binding of norepinephrine to the α1 receptors on the membrane of the vascular smooth muscle cells [32]. The resulting vasodilation after the administration of doxazosin suggests the sympathetic hyperactivity of the present animal model. However more studies should be performed to support this theory.

Nowadays, measurement of BP by radiotelemetry has been described and validated for many laboratory animal species. This technique eliminates the need for tethering or restraining the animals, which introduce stress and artifacts during data sampling [33]. However, the use of this method is limited due to its prohibitive cost, especially when not used frequently. Different direct methods under anesthesia have also been proposed to assess the BP, such as pigtail catheter placed in the aorta [12] or in the femoral artery [13]. There is, however, controversy regarding the values of BP when the animal is under anesthesia, indicating a 20% loss in mean arterial pressure when the animal is under anesthesia [34]. For this reason, we tried direct arterial BP monitoring in a conscious state by the placement of a BP measurement indwelling catheter. However, the high mortality associated with this procedure due to infections, arterial thrombosis and hemorrhages forces us to modify the present protocol or to use other monitoring systems in future studies.

In the present model, the reduction in BP was related to the nerve injury identified by the reduced immunohistochemical expression of TH, which is considered key evidence of a proper RF nerve ablation [19]. In this sense, the non-response of one animal was associated with no damaged nerves. Possible explanations include an incomplete denervation given the heterogeneous distribution of sympathetic nerves and the inter-individual variability. The relationship between the reduction in BP and percentage of damaged nerves was observed more clearly in the distal ablations. Human histological studies as well as preclinical animal studies indicate that the maximal numbers of nerves are located around the proximal and middle segments of the renal artery, whereas lower numbers of nerves are located around distal structures. However, the mean distance of the sympathetic afferent and efferent nerves to the lumen of the renal arteries is shorter in the distal arterial segments [28]. We hypothesize that the effectiveness of radiofrequency RDN is enhanced when the procedure is focused on the distal area, likely because of the proximity of the nerves to the treated artery, which is in line with recently published reports [35,36,37].

One of the major unsolved issues of the procedure is how to monitor treatment success intraprocedurally. In the present study, animals with reduced BP showed NE gradient reduction and impedance drop, although there was not a clear relationship between the magnitude of NE reduction/impedance drop and the reduction in BP. However, the case of animal # 3 suggests a potential working hypothesis: no nerve damage was observed in the case when the impedance drop was below 10% during the procedure; in addition, NE gradient did not decrease (in fact, it increased) and BP response was flat. Be it a technical failure in the RF RDN procedure or an individual abnormal response, the absence of all the studied responses is interesting.

Finally, the RF procedure displayed a high safety with none-mild inflammatory response, complete vascular endothelialization without vascular thrombosis, hemorrhage or dissections, similar to the one described in clinical and preclinical trials using radiofrequency energy [38,39,40]. However, long-term follow-up in a larger set of animals is required before statements on the safety profile can be made.

### Study Limitations

The main limitation of this study was that the creation of the model requires a learning curve and the placement of the blood pressure catheter measurement produces significant technical difficulties and has a direct impact on the clinical outcomes of the animals. We believe that a combination of our suggested animal model with a telemetric measuring technique would decrease infection rates. Another limitation is related to the physiological responses of primary hyperaldosteronism (PHA), that is not the most common form of clinical hypertension. However, the decrease in blood pressure following surgical denervation of a DOCA swine model [33] supports the potential use for this model in catheter-based RDN studies. Third, the sample size is small, especially taking into account the high attrition rate. Most of the observations should be confirmed in larger experimental studies.

## 5. Conclusions

The data obtained in this preliminary study suggest that the DOCA large animal model could be used as an experimental platform of reproducible, long term maintenance of arterial hypertension. We believe that further investigation with proper animal models, such as the one presented in this study, is critical for the advancement of this therapy. Ablations performed in the distal region might improve the effectiveness of radiofrequency RDN. This observation seems to confirm the importance of anatomic and procedural parameters when performing RDN and deserves further investigation in clinical studies. However, the high mortality should be addressed in the next iterations of this model.

## Figures and Tables

**Table 1:** Percentage of damaged nerves by tyrosine hydroxylase immunostaining (score ≤ 2).

Animals	Number of Damaged Nerves/Number of Nerves (%)		
	Proximal	Medial	Distal
1	46/65 (70.8%)	55/104 (52.9%)	88/152 (57.9%)
2	35/48 (72.9%)	87/129 (67.4%)	16/43 (37.2%)
3	5/35 (14.3%)	0/31 (0%)	0/66 (0%)
4	42/83 (50.6%)	39/69 (56.5%)	52/82 (63.41%)

**Table 2:** Procedural characteristics in denervated animals.

Animal	# Ablation Points	# Ablation Points for 2 min	Impedance Drop (%)	Maximum Temp (°C)	Mean Temp (°C)	Maximum Energy Output (W)	NE Gradient
1	13	13	12.4	59.4	54.3	8	−9
2	10	9	16.6	69.7	57.2	8	−20.3
3	19	12	6.5	74.9	54.1	8	+157
4	10	8	10	69.4	51.6	8	−6

#, number; min, minutes; Temp, temperature; NE, norepinephrine.
